# Intraspecific variation in phenotypic and phylogenetic features among *Pratylenchus penetrans* isolates from Wisconsin, USA

**DOI:** 10.21307/jofnem-2020-102

**Published:** 2020-11-24

**Authors:** Kanan Saikai, Ann E. MacGuidwin

**Affiliations:** Department of Plant Pathology, University of Wisconsin–Madison, 484 Russell Laboratories, 1630 Linden Dr., Madison, WI, 53706

**Keywords:** Haplotype, Morphology, Root-lesion nematode

## Abstract

*Pratylenchus penetrans* is a common and important agricultural pest in Wisconsin, a USA state with a diverse agriculture. We compared populations from around the state to each other and to data published for populations around the world to gain insight on the variability of features important for identification of this cosmopolitan species. Thirteen isolates from samples collected in soybean fields in ten Wisconsin counties were established in monoxenic cultures. Analysis of morphological features revealed the least variable feature for all isolates collectively was vulva percentage. Features less variable within than among isolates were body width, lip region height, and stylet length. Some isolates showed only the smooth tail tip phenotype and others had a mix of smooth and annulated tail phenotypes. A suite of features provided sufficient pattern to group isolates into four clusters according to hierarchical agglomerative clustering and canonical discriminative analyses, but not with enough distinction to be useful for classification. Haplotype analysis based on the COI mitochondrial gene of the 13 cultured isolates, 39 Wisconsin field populations, and published sequences representing five additional USA states and six countries revealed 21 haplotypes, 15 of which occurred in Wisconsin. Ten haplotypes represented in Wisconsin were shared with populations from Europe, South America, Africa, or Asia. Five haplotypes were unique to Wisconsin, six were unique to The Netherlands, and one was unique to Japan suggesting that even more COI diversity will be revealed when more COI sequences for *P. penetrans* become available. The maximum pairwise sequence variation was 6% and the SNPs did not alter amino acids, indicating cryptic biodiversity within the species worldwide. The cosmopolitan to localized scale of distribution of COI haplotypes could be due to frequent and ongoing dispersal events, facilitated by life history traits and the broad host range of *P. penetrans*. Regions of diverse agriculture, like Wisconsin, show promise for studying this important pest and our study confirms the utility of the COI mtDNA gene for studying variation within a species.

*Pratylenchus penetrans* (Cobb, 1917) is a cosmopolitan species reported from 69 countries and every continent except Antarctica (EPPO, 2020). Plant damage due to parasitism by *P. penetrans* has been documented across the plant kingdom with the most severe impact occurring in temperate climates. In Wisconsin, a state in the North Central USA with a diverse agriculture, *P. penetrans* is a common pest of fruit, vegetable, grain, and forage crops.

Identification of *P. penetrans* is supported by multiple species keys for the genus *Pratylenchus* (Loof, 1960; Corbett, 1970; Handoo and Golden, 1989; Ryss, 2002; Castillo and Vovlas, 2007). Consensus morphological characters are three lip annuli, round spermatheca, presence of males, smooth tail tip, and four incisures in the lateral field. Morphological measurements and indices of morphological features are also important, particularly the proportional position of the vulva (V%) and relative length of the esophagus and tail.

Morphological features and measurements of *P. penetrans* have been recorded many times since its original description as *Tylenchus penetrans* by Cobb (1917). We found records of four populations from the USA and 32 populations from other countries (Sher and Allen, 1953; Taylor and Jenkins, 1957; Loof, 1960; Román and Hirschmann, 1969; Tarte and Mai, 1976a; Vovlas and Troccoli, 1990; Townshend, 1991; Troccoli et al., 1992; Mekete et al., 2011; Mokrini et al., 2016; Janssen et al., 2017; Ozbayrak, 2019; Rusinque et al., 2020). The range in values for quantitative features barely overlaps among some populations. Intraspecific variation in tail morphology was noted by Taylor and Jenkins (1957), Román and Hirschmann (1969), Tarte and Mai (1976a), Townshend (1991) and Rusinque et al. (2020), including the observation that tail features as well as body size could be modified by environmental factors such as plant host and culture media (Tarte and Mai, 1976a; Townshend, 1991).

There is consensus that nematode identification today should be supported by molecular analyses so sequence data are rapidly accumulating for multiple gene fragments including the cytochrome c oxidase subunit1 (COI) mitochondrial gene (Janssen et al., 2017), rDNA internal transcribed spacers (De Luca et al., 2011), D2/D3 expansion region of 28S rDNA, and 18S rDNA (Subbotin et al., 2008). Janssen et al. (2017) used a combination of morphological and sequence data of COI to successfully distinguish species among the Penetrans group of *Pratylenchus* spp; *P. penetrans, P. fallax, P. convallariae, P. dunensis, P. pinguicaudatus, P. brachyurus, P. arlington*, and *P. oleae.*

Intraspecific genetic variation among *P. penetrans* populations was addressed recently by two studies (Mokrini et al., 2016; Janssen et al., 2017). Mokrini et al. (2016) found relatively little variation among 13 Moroccan isolates of *P. penetrans* for the D2/D3 expansion region of 28S rDNA. Janssen et al. (2017) reported 16 haplotypes based on COI for *P. penetrans* isolates collected around the world. Their analysis revealed 14 of the 16 haplotypes were represented in The Netherlands. Three of the 16 haplotypes were shared by populations from The Netherlands, Africa, and Asia. North America was represented by only one population of *P. penetrans* and it was the only member of one haplotype. The study by Janssen et al. (2017) also revealed cryptic biodiversity within *P. penetrans,* as morphologically indistinguishable populations from The Netherlands were assigned to different haplotypes.

We recently surveyed Wisconsin for species of *Pratylenchus* with males to determine the prevalence of *P. penetrans* in crop rotations including soybean. That project (Saikai, 2019) resulted in first reports of *P. fallax* (Saikai et al., 2019) and *P. alleni* (Saikai and MacGuidwin, 2019) in Wisconsin, but also showed the vast majority of populations with *Pratylenchus* males to be *P. penetrans* based on sequence data from 28S rDNA, 18S rDNA, and a species-specific primer. Our objectives in this study were (i) to assess the extent of intraspecific variability in *P*. *penetrans* in Wisconsin by comparing 13 populations of *P*. *penetrans* for morphological variation and 52 populations for sequence variation in the COI mitochondrial gene, and (ii) to determine the extent of diversity in COI for *P*. *penetrans* by combining our data with published COI sequences from populations around the world in phylogenetic and haplotype analyses.

## Materials and methods

### Nematode isolates

Nematodes were recovered from soil samples collected from 11 farms in nine Wisconsin counties for a commodity-sponsored testing program. The samples were randomly selected from a pool of stored samples that were known to contain *Pratylenchus* males. Nematodes were extracted from root fragments sieved from the soil and incubated in water for 48 hr. One female from the incubated fraction was placed on an excised root culture of ‘IO Chief’ sweet corn (*Zea mays* L.) grown in Gamborg’s B5 media, with the assumption that insemination occurred in the field. Cultures were maintained in the dark at 24°C and transferred to new Gamborg’s B5 media every five months to increase nematodes. Each population was verified as *P. penetrans* by morphology, the species-specific primer *PPEN* (Al-Banna et al., 2004), and matching sequences of the D2/D3 expansion region of 28S rDNA to accessions reported in GenBank. Two additional isolates of *P. penetrans* in culture for more than 25 years (Portage1 and Portage2), originally extracted from potato, were included in the study for a total of 13 isolates, each named for the county of origin (10 counties total).

### Morphological characterization of isolates

Nematodes from at least three Petri dishes were harvested and pooled for morphological characterization, given the reported sensitivity of some features to environmental conditions (Tarte and Mai, 1976a). Fresh specimens were mounted following the protocol of Eisenback (2012), visualized using differential interference contrast optics, and measured and photographed using NIS-Elements AR Software on a Nikon Eclipse Ti-E inverted microscope (Nikon Instruments, Melville, NY). Tail terminus morphology, tail shape, and spermatheca shape were recorded. Data were collected from a minimum of 25 females of each isolate, except for Chippewa2 (*n* = 18 females), for 26 morphometric and morphological variables. The same morphometrics, except for those that are specific to females, were measured for males of each isolate but only spicule length and gubernaculum are listed in this study. The following indices were derived: V% (anterior end to vulva length/total body length), and *a* (total body length/maximum body width), *b* (total body length/esophageal length), *c* (total body length/tail length), *b′* (total body length/distance from anterior end to the end of esophageal glands), and *c′* (tail length/body width at anus). Tail shape and tail tip appearance were assigned according to the categories used by Frederick and Tarjan (1989). The coefficient of variation (CV) was computed for each morphological variable by isolate and for pooled data representing all isolates.

### Phenotype analyses of isolates

Intraspecific variation in the morphometrics among the *P. penetrans* isolates were evaluated with Hierarchical Agglomerative clustering (HAC) and Canonical Discriminant analysis (CDA) for the following female features: L, V%, ratios of *a*, *b*, *c*, *c′*, maximum body width, esophageal length, length of anterior end to excretory pore, lip region height, stylet length, DGO, PUS, body width at anus, tail length, vulva to anus distance, lateral incisures, and labial incisures. Based on high correlation coefficients with other features, the *b′* ratio, length of head end to posterior gland and body width at vulva were excluded from phenotype analyses. The shape of the tail and annulation of the tail terminus were included as binary variables: subhemispherical tail = 0, not subhemispherical tail = 1; smooth tip = 0, crenate tip = 1.

For HAC, Euclidean distance coefficients were computed on normalized mean values to evaluate the (dis)similarity between each pair of isolates using the ‘dist’ function with the Euclidean method option in the ‘STATS’ R package (R Core Team, 2020). The normalized means were computed by $X\prime =\frac{X-X_{\min}}{X_{\max}-X_{\min}},$ so that all the morphometrics ranged between 0 and 1. HAC was performed using the ‘hclust’ function of the same R package with Ward’s method. The optimum number of clusters was determined using the Elbow and Silhouette methods.

Canonical discriminant analysis (CDA) of the clusters suggested by HAC was performed using the CANDISC procedure in SAS (SAS Institute, Cary, NC) to determine which variables discriminated clusters. Univariate statistics (ANOVA) were also conducted for each morphometric variable to test equality of means among the 13 isolates.

### COI sequencing

**Figure 1:**
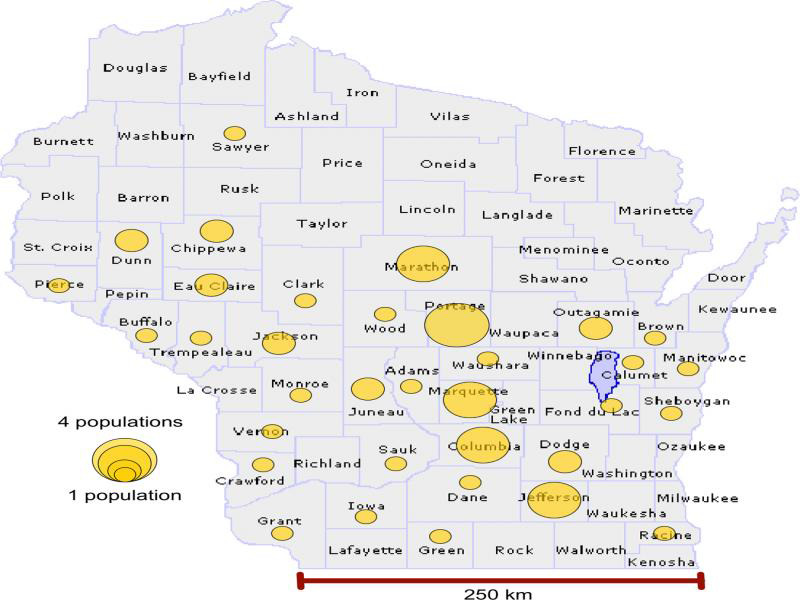
Map of Wisconsin, USA, marked with circles that represent the number of isolates and field populations sampled for haplotype network analysis per county.

Nematodes from the 13 cultured isolates and 39 field populations collected from 39 farms in 27 Wisconsin counties were characterized molecularly for mitochondrial cytochrome c oxidase subunit 1 (COI) (Fig. 1). The 52 populations were named for the county of origin and multiple populations from the same country were distinguished by numbers and individual specimens by letters (Table 3). Nematode DNA was extracted using DNeasy Blood and Tissue Kit (Qiagen, Valencia, CA). The DNA extracts were stored at −20°C if PCR was not conducted immediately. Five microliters of DNA were suspended in a 25 μl reaction volume composed of 2.5 μl ddH_2_O, 2.5 μl of forward and reverse primers, and 12.5 μl of GoTaq Green Master Mix x2 (Promega, Madison, WI). The primer used for COI was forward *JB3* (5′-TTTTTTGGGCATCCTGAGGTTTAT-3′) and reverse *JB4.5* (5′-TAAAGAAAGAACATAATGAAAATG-3′) (Derycke et al., 2010). PCR cycling conditions were: 94°C for 5 min, 5 cycles of denaturation at 94°C for 30 sec, annealing at 54°C for 30 sec and temperature decreasing with 1°C for each cycle, and extension at 72°C for 30 sec followed by 35 cycles of 94°C for 30 sec, 50°C for 30 sec, and 72°C for 30 sec, and a final extension of 10 min at 72°C. Five micro liters of the PCR products were resolved by electrophoresis on 1.0% agarose gel mixed with 2.5 μl of SYBR Safe DNA Stain (Invitrogen, Waltham, WA) at 70 V for 1 hr. The presence of DNA in the PCR products was confirmed by Gel Logic 200 Imaging System (Kodak alaris, Rochester, NY). The crude PCR products were sent to Functional Biosciences (Madison, WI) for purification and Sanger-sequencing.

**Table 3.** Information for origin and hosts of the *Pratylenchus penetrans* isolates and populations used for this study, COI haplotype group assigned in this study, and GenBank accession numbers for D2/D3 expansion region of 28S and COI sequences.

				GenBank Accession number	
Locality	Specimen name	Planted crop	Haplotype group	COI	D2/D3 of 28S rRNA
USA, Wisconsin, Brown Co.	Brown-a	corn	Pp1	MT527067	MT528159
*USA, Wisconsin, Buffalo Co.*	*Buffalo-a*	*soybean*	Pp1	*MN453207*	*MN251253*
*USA, Wisconsin, Calumet Co.*	*Calumet-a*	*soybean*	Pp1	*MN453210*	*MN251255*
*USA, Wisconsin, Chippewa Co.*	*Chippewa2-a*	*soybean*	Pp1	*MN453213*	*MN251258*
USA, Wisconsin, Columbia Co.	Columbia1-a	corn	Pp1	MT527044	MT528189
USA, Wisconsin, Columbia Co.	Columbia3-a	corn	Pp1	MT527077	MT528192
USA, Wisconsin, Crawford Co.	Crawford-b	–	Pp1	MT527055	MT528196
USA, Wisconsin, Dunn Co.	Dunn1-b	soybean	Pp1	MT527024	MT528199
USA, Wisconsin, Eau Claire Co.	Eau Claire1-a	soybean	Pp1	MT527073	MT528203
USA, Wisconsin, Eau Claire Co.	Eau Claire2-a	soybean	Pp1	MT527059	MT528205
*USA, Wisconsin, Grant Co.*	*Grant-a*	*soybean*	Pp1	*MN453211*	*MN251256*
*USA, Wisconsin, Iowa Co.*	*Iowa-a*	*soybean*	Pp1	*MN453217*	*MN251263*
USA, Wisconsin, Jackson Co.	Jackson1-a	soybean	Pp1	MT527038	MT528171
USA, Wisconsin, Jackson Co.	Jackson2-a	soybean	Pp1	MT527053	MT528208
USA, Wisconsin, Jefferson Co.	Jefferson2-a	soybean	Pp1	MT527015	MT528173
USA, Wisconsin, Jefferson Co.	Jefferson3-a	corn	Pp1	MT527085	MT528210
USA, Wisconsin, Juneau Co.	Juneau1-a	–	Pp1	MT527037	MT528175
USA, Wisconsin, Manitowoc Co.	Manitowoc-a	corn	Pp1	MT527045	MT528215
*USA, Wisconsin, Marathon Co.*	*Marathon1-a*	*soybean*	Pp1	*MN453215*	*MN251261*
USA, Wisconsin, Marathon Co.	Marathon3-a	–	Pp1	MT527035	MT528176
*USA, Wisconsin, Marquette Co.*	*Marquette1-a*	*soybean*	Pp1	*MN453214*	*MN251259*
USA, Wisconsin, Marquette Co.	Marquette2-a	corn	Pp1	MT527090	MT528216
USA, Wisconsin, Monroe Co.	Monroe-b	–	Pp1	MT527019	MT528178
USA, Wisconsin, Outagamie Co.	Outagamie1-a	soybean	Pp1	MT527089	MT528219
USA, Wisconsin, Outagamie Co.	Outagamie2-a	soybean	Pp1	MT527070	MT528220
USA, Wisconsin, Pierce Co.	Pierce-a	soybean	Pp1	MT527042	MT528222
USA, Wisconsin, Portage Co.	Portage3-a	soybean	Pp1	MT527074	MT528223
USA, Wisconsin, Portage Co.	Portage4-b	–	Pp1	MT527021	MT528182
USA, Wisconsin, Sauk Co.	Sauk-a	soybean	Pp1	MT527048	MT528227
USA, Wisconsin, Sawyer Co.	Sawyer-a	soybean	Pp1	MT527064	MT528230
USA, Wisconsin, Vernon Co.	Vernon-a	soybean	Pp1	MT527050	MT528232
USA, Wisconsin, Waushara Co.	Waushara-c	kidney beans	Pp1	MT527017	MT528188
USA, Nebraska, Otoe Co.	–	apple	Pp1	MK877987^b^	–
USA, Nebraska, Phelps Co.	–	corn	Pp1	MK877993^b^	–
Japan, Chiba	CA193^a^	wild carrot	Pp1	KY817024^a^	EU130857^c^
The Netherlands, Zoetermeer	T181^a^	grasses	Pp1	KY817007^a^	
Colombia, San Vicente	T666^a^	cape gooseberry	Pp1	KY816982^a^	KY828351^a^
Colombia, San Vicente	T677^a^	cape gooseberry	Pp1	KY816981^a^	KY828350^a^
Colombia, San Vicente	T678^a^	cape gooseberry	Pp1	KY816980^a^	KY828349^a^
Colombia, San Vicente	T679^a^	cape gooseberry	Pp1	KY816979^a^	
The Netherlands, Wemeldinge	T725^a^	sweet cherry	Pp1	KY816949^a^	
The Netherlands, Nagele	V3F^a^	apple	Pp1	KY916940^a^	KY828346^a^
The Netherlands , Meijel	V4B^a^	apple	Pp1	KY816939^a^	KY828345^a^
The Netherlands, Wemeldinge	V8A^a^	apple	Pp1	KY816936^a^	KY828342^a^
*USA, Wisconsin, Marathon Co.*	*Marathon2-a*	*soybean*	*Pp2*	*MN453219*	*MN251265*
Japan, Aichi	CA85^a^	wild cabbage	Pp3	KY817021^a^	EU130860^c^
USA, Wisconsin, Clark Co.	Clark-b	corn	Pp4	MT527033	MT528161
USA, Wisconsin, Marquette Co.	Marquette3-a	soybean	Pp4	MT527079	MT528218
USA, Wisconsin, Outagamie Co.	Outagamie2-b	soybean	Pp4	MT527071	MT528221
USA, Alaska	–	peony	Pp4	MK877984^b^	–
The Netherlands, Stramproy	T716^a^	sweet cherry	Pp5	KY816958^a^	
USA, Wisconsin, Monroe Co.	Monroe-a	–	Pp6	MT527018	MT528177
*USA, Wisconsin, Portage Co.*	*Portage2-a*	*soybean*	Pp6	*MN453205*	*MN251267*
USA, Minnesota	–	corn	Pp6	MK877982^b^	–
The Netherlands, Stramproy	T715^a^	sweet cherry	Pp6	KY816959^a^	
USA, Wisconsin, Clark Co.	Clark-c	corn	Pp7	MT527033	MT528161
USA, Wisconsin, Dunn Co.	Dunn2-a	soybean	Pp7	MT527054	MT528202
USA, Wisconsin, Racine Co.	Racine-a	soybean	Pp7	MT527062	MT528224
The Netherlands, Meijel	V1B^a^	apple	Pp7	KY816942^a^	KY828348^a^
USA, Wisconsin, Adams Co.	Adams-a	–	Pp8	MT527029	MT528157
*USA, Wisconsin, Portage Co.*	*Portage1-a*	*soybean*	Pp8	*MN453204*	*MN251266*
Rwanda, Nyakirbia	T172^a^	pear	Pp8	KY817009^a^	KY828356^a^
The Netherlands, Apeldorn	T293^a^	pear	Pp8	KY816992^a^	KY828353^a^
The Netherlands, Sambeek	T686^a^	apple	Pp8	KY816976^a^	
The Netherlands, Sambeek	T687^a^	apple	Pp8	KY816975^a^	
The Netherlands, Schimmert	T699^a^	plum	Pp8	KY816972^a^	
The Netherlands, Kloetinge	T723^a^	sweet cherry	Pp9	KY816951^a^	
USA, Wisconsin, Clark Co.	Clark-a	corn	Pp10	MT527034	MT528160
USA, Wisconsin, Columbia Co.	Columbia2-a	soybean	Pp10	MT527078	MT528191
USA, Wisconsin, Crawford Co.	Crawford-a	–	Pp10	MT527056	MT528195
USA, Wisconsin, Dodge Co.	Dodge1-a	soybean	Pp10	MT527025	MT528162
USA, Wisconsin, Dodge Co.	Dodge2-a	soybean	Pp10	MT527041	MT528163
USA, Wisconsin, Dodge Co.	Dodge2-b	soybean	Pp10	MT527080	MT528164
USA, Wisconsin, Eau Claire Co.	Eau Claire2-b	soybean	Pp10	MT527058	MT528206
USA, Wisconsin, Fond du Lac Co.	Fond du Lac-a	wheat	Pp10	MT527084	MT528165
USA, Wisconsin, Green Co.	Green-a	–	Pp10	MT527032	MT528169
USA, Wisconsin, Jackson Co.	Jackson1-b	soybean	Pp10	MT527039	MT528170
USA, Wisconsin, Jefferson Co.	Jefferson1-a	corn	Pp10	MT527040	MT528172
USA, Wisconsin, Jefferson Co.	Jefferson2-b	soybean	Pp10	MT527026	MT528174
USA, Wisconsin, Juneau Co.	Juneau2-a	soybean	Pp10	MT527087	MT528212
USA, Wisconsin, Monroe Co.	Monroe-c	–	Pp10	MT527051	MT528179
USA, Wisconsin, Portage Co.	Portage4-a	–	Pp10	MT527020	MT528183
USA, Wisconsin, Sauk Co.	Sauk-b	soybean	Pp10	MT527047	MT528228
USA, Wisconsin, Trempeleau Co.	Trempeleau-a	–	Pp10	MT527028	MT528185
USA, Wisconsin, Waushara Co.	Waushara-b	kidney beans	Pp10	MT527036	MT528187
*USA, Wisconsin, Wood Co.*	*Wood-a*	*soybean*	Pp10	*MN453208*	*MN251254*
USA, Idaho	–	potato	Pp10	MK877988^b^	–
USA, Wisconsin, Waushara Co.	Waushara-a	kidney beans	Pp11	MT527016	MT528186
*USA, Wisconsin, Sheboygan Co.*	*Sheboygan-a*	*soybean*	*Pp12*	*MN453212*	*MN251257*
*USA, Wisconsin, Chippewa Co.*	*Chippewa1-a*	*soybean*	*Pp13*	*MN453206*	*MN251252*
USA, Wisconsin, Dunn Co.	Dunn1-a	soybean	Pp14	MT527082	MT528200
USA, California, Stanislaus Co.	CA91^a^	cowpea	Pp14	KY817023^a^	EU130862^c^
USA, Wisconsin, Dane Co.	Dane-b	soybean	Pp15	MT527068	MT528198
France, Britany	CA192^a^	apple	Pp15	KY817022^a^	KY828242^a^
Ethiopia, Shashamane	T225^a^	corn	Pp15	KY817001^a^	KY828354^a^
The Netherlands, Hei en Boeicop	T296^a^	pear	Pp15	KY816990^a^	–
The Netherlands, Vredepeel	T44^a^	wild carrot	Pp15	KY817020^a^	KY828359^a^
The Nethelands, Schimmert	T697^a^	plum	Pp15	KY816974^a^	–
The Netherlands, Kloetinge	T720^a^	sweet cherry	Pp15	KY816954	–
The Netherlands, St. Oedenrode	T730^a^	grape vine	Pp15	KY816944^a^	–
The Netherlands, Baarlo	V3A^a^	apple	Pp15	KY816941^a^	KY828347^a^
The Netherlands, Wemeldinge	T726^a^	sweet cherry	Pp16	KY816948^a^	
USA, Wisconsin, Adams Co.	Adams-b	–	Pp17	MT527066	MT528158
USA, Wisconsin, Sawyer Co.	Sawyer-b	soybean	Pp17	MT527065	MT528229
USA, Wisconsin, Columbia Co.	Columbia1-b	corn	Pp18	MT527043	MT528190
The Netherlands, Stramproy	T714^a^	sweet cherry	Pp18	KY816960^a^	–
The Netherlands, Stramproy	T713^a^	sweet cherry	Pp19	KY816961^a^	–
USA, Wisconsin, Dane Co.	Dane-a	soybean	Pp20	MT527069	MT528197
USA, Wisconsin, Marquette Co.	Marquette2-b	corn	Pp20	MT527091	MT528217
Rwanda, Nyakirbia	T143^a^	onion	Pp20	KY817013^a^	KY828357^a^
Rwanda, Nyakiriba	T144^a^	onion	Pp20	KY817012^a^	
Rwanda, Nyakiriba	T145^a^	onion	Pp20	KY817011^a^	
The Netherlands, Arkel	T184^a^	pear	Pp20	KY817005^a^	
Rwanda, Bushoki	T200^a^	potato	Pp20	KY817004^a^	KY828355^a^
The Netherlands, Hei en Boeicop	T295^a^	pear	Pp20	KY816991^a^	KY828352^a^
The Nethelands, Schimmert	T698^a^	plum	Pp20	KY816973^a^	
The Netherlands, Kloetinge	T721^a^	sweet cherry	Pp20	KY816953^a^	
The Netherlands, Wemeldinge	T724^a^	sweet cherry	Pp20	KY816950	
The Nethelands, Zoetermeer	T132^a^	grasses	Pp21	KY817015^a^	KY828358^a^
The Netherlands, Kloetinge	T722^a^	sweet cherry	Pp21	KY816952	

**Notes:** The Wisconsin isolates used for phenotypic and phylogenetic studies are in italic. ^a^ Janssen et al. (2017); ^b^ Ozbayrak et al. (2019); ^c^Subbotin et al. (2008).

The DNA sequences were edited with Finch TV (Geospiza Inc, Seattle, WA), and aligned with MUSCLE in MEGA 7 (Kumar et al., 2016) using default parameters with the gap opening penalty set at 15 and the gap extension penalty at 6.66. Alignments were further adjusted by inspection. A complemented sequence was constructed by overlapping sequence reads with forward and reverse primers for each specimen. Basic Local Alignment Search Tool (BLAST) from the National Center for Biotechnology Information (NCBI) was used to compare the complemented sequences to the GenBank sequence database. Sequences were deposited to GenBank and assigned accession numbers (Table 3).

### Haplotype network and phylogenetic analyses

Redundant COI sequences among multiple individuals sequenced from the same population were dropped, leaving 72 sequences for the haplotype analysis, 13 from cultured isolates, and 59 from field populations. In total, 48 published sequences from populations in other USA states (Ozbayrak et al., 2019) and the populations used in the Janssen et al.’s (2017) study were also included for a total of 120 sequences used in the analyses. The haplotype network was calculated and visualized using TCS network in the software PopART Version 1.7. Geographical origin of the sequences was used as trait information in the analysis.

For phylogenetic analysis, maximum-likelihood (ML) trees were constructed based on the aligned sequences of COI using the default program settings in MEGA 7 with 1000 bootstrap replications. The best DNA model of the nucleotide substitution tool option in MEGA 7 selected the Hasegawa-Kishino-Yano model with a gamma distribution and proportion of invariable sites (HKY + G + I) for COI. The COI sequences of all the Wisconsin populations and CA85, T716, T723, T726, T713, T132 as well as the other species in the Penetrans group and *P. crenatus* were included in the analysis. *Meloidogyne hapla* (JX683719) and *M. camelliae* (KM887147) were included as outgroup taxa per the study by Janssen et al. (2017).

## Results

### Morphology and morphometrics of isolates

All 13 Wisconsin isolates possessed the diagnostic characters of the species; three lip annuli, round spermatheca filled with sperm, four incisures in the lateral field, and a majority of specimens had a smooth tail tip (Tables 1 and 2). Stylet basal knobs were usually round but sometimes cupped anteriorly. The CV for the pooled data of all isolates was greatest for PUS (24.8%) followed by lip region height (17.4%) and *c′* (17.3%). The morphometrics with the smallest variability were V% (1.8%), stylet length (6.0%), and length of head end to the end of the esophageal gland (9.0%). The maximum value for the CV for individual isolates was lower than the pooled CV for body width, lip region height, and stylet length.

**Table 1.** Mean and range of morphometrics and morphological features of *Pratylenchus penetrans* isolates from Portage, Marathon, and Chippewa counties in Wisconsin.

	Portage 1	Portage 2	Marathon 1	Marathon 2	Chippewa 1	Chippewa 2
*L*^a^	675 (602-832)	637 (544-748)	595 (513-682)	628 (507-702)	594.8 (494-694)	630 (564-704)
V%	80.3 (77.5-82.5)	79.0 (75.0-82.0)	78.8 (75.6-81.1)	80.9 (78.9-86.6)	80.2 (77.3-81.9)	80.7 (78.5-82.3)
*a*	26.4 (22.1-33.2)	27.9 (24.8-32.0)	25.9 (19.9-30.3)	23.6 (20.2-25.7)	26.1 (20.6-28.7)	23.0 (18.4-28.2)
*b*	5.62 (4.37-6.74)	6.11 (5.11-7.53)	5.69 (4.81-6.93)	6.09 (5.14-7.20)	5.18 (4.15-5.18)	5.43 (4.89-6.00)
*c*	18.7 (15.1-23.1)	21.4 (17.3-26.7)	19.2 (16.3-24.8)	19.8 (14.4-31.2)	18.8 (15.4-24.2)	20.4 (16.4-22.8)
*b′*	4.86 (3.89-5.63)	5.33 (4.39-8.76)	4.81 (4.17-5.78)	5.15 (4.34-6.09)	4.39 (3.64-5.31)	4.68 (4.26-5.10)
*c′*	2.44 (1.93-3.06)	2.39 (1.99-2.88)	2.43 (1.67-2.94)	2.09 (1.42-2.86)	1.67 (1.24-2.90)	1.97 (1.71-2.31)
Maximum body width	25.8 (21.3-29.4)	22.9 (19.3-27.5)	23.2 (19.0-29.3)	26.7 (21.4-33.9)	22.7 (19.1-27.0)	27.7 (21.0-33.7)
Esophageal length	121 (102-142)	103 (72-128)	105 (91-125)	104 (83-121)	116 (102-133)	116 (97-138)
Head end to posterior gland	140 (122-160)	121 (84-145)	124 (109-144)	123 (101-141)	134 (120-152)	135 (115-157)
Anterior end to excretory pore	97 (80-108)	92 (67-106)	91 (77-101)	90 (81-98)	89 (80-99)	93 (84-112)
Lip region height	2.47 (2.07-3.10)	2.89 (2.4-4.07)	2.41 (1.87-2.92)	2.62 (2.09-3.15)	2.44 (1.94-3.43)	2.36 (2.04-2.77)
Stylet	14.8 (13.9-16.2)	14.3 (13.4-15.4)	15.5 (14.1-17.5)	14.8 (13.3-16.3)	16.8 (15.3-17.9)	15.3 (13.9-16.4)
DGO	2.67 (2.13-3.51)	2.93 (2.26-3.87)	2.92 (2.08-3.54)	2.46 (1.75-3.48)	2.73 (2.15-3.71)	2.61 (1.89-3.42)
Body width at vulva	22.2 (17.6-25.9)	20.0 (17.0-22.5)	20.2 (17.8-22.4)	23.6 (17.9-28.4)	20.2 (17.9-25.1)	23.4 (18.9-27.9)
PUS	18.5 (15.1-22.4)	14.4 (9.4-21.7)	16.4 (12.1-21.8)	16.5 (12.4-20.8)	21.4 (14.1-35.0)	18.3 (14.2-24.1)
Body width at anus	15.05 (13.5-17.0)	14.6 (12.7-16.9)	13.8 (12.2-15.3)	15.7 (12.8-18.3)	14.1 (12.5-16.5)	15.9 (13.1-19.8)
Tail length	36.6 (30.8-52.2)	34.8 (28.6-40.6)	33.5 (20.3-44.0)	35.6 (22.3-41.8)	31.7 (22.9-42.0)	31.2 (27.0-45.5)
Vulva to anus distance	95 (79-119)	94 (74-114)	91 (81-105)	89 (63-108)	87 (67-110)	91(79-107)
Lateral incisures	4 with striation	4	4	4	4 with striation	4
Labial annules	3	3	3	3	3	3
Tail tip^b^	SMO	SMO	SMO	SMO	SMO	SMO
Tail shape^c^	SHM	SHM	SHM	SHM	SHM	SHM
Spermatheca	Round	Round	Round	Round	Round	Round
Spicule	14.3 (11.7-17.5)	14.8 (12.0-16.6)	15.3 (12.4-17.2)	14.7 (11.7-18.2)	16.1 (13.2-19.6)	15.6 (10.9-19.2)
Gubernaculum	4.09 (2.71-5.71)	3.92 (2.94-5.67)	4.86 (3.87-5.99)	3.62 (3.02-4.22)	4.49 (3.50-5.94)	3.56 (2.43-4.95)

**Notes:** ^a^ *L* = total body length; V% = vulva% from the anterior end; *a* = *L*/maximum body width; *b* = *L*/esophageal length; *c* = *L*/tail length; *b′* = *L*/anterior end to end of esophageal gland; *c′* = tail length/body width at anus; ^b^SMO = smooth; after Federick and Tarjan (1989); ^c^SHM = subhemisphericacl; after Federick and Tarjan (1989).

**Table 2.** Mean and range of morphometrics and morphological features of the *Pratylenchus penetrans* isolates from Calumet, Sheboygan, Wood, Marquette, Buffalo, Grant, and Iowa counties in Wisconsin.

	Calumet	Sheboygan	Wood	Marquette 1	Buffalo	Grant	Iowa
*L*^a^	603 (497-844)	624 (556-728)	605 (541-717)	603 (518-749)	548 (465-608)	588 (495-704)	701 (631-818)
V%	79.9 (76.0-82.4)	79.7 (77.6-81.7)	79.2 (74.8-81.0)	80.0 (77.3-81.8)	80.9 (78.2-83.9)	80.8 (78.3-85.2)	79.7 (77.4-81.9)
*a*	28.7 (24.2-38.5)	28.1 (25.0-30.4)	26.2 (20.9-30.3)	24.6 (20.7-29.5)	25.7 (22.7-29.2)	28.2 (22.6-31.9)	24.1 (21.1-27.7)
*b*	5.39 (4.44-6.11)	5.69 (4.77-7.10)	5.33 (4.59-6.38)	5.68 (4.42-7.22)	5.20 (4.46-6.21)	5.12 (4.34-6.11)	6.44 (5.36-7.56)
*c*	18.8 (13.9-23.7)	20.9 (16.1-33.2)	23.0 (19.2-31.0)	20.1 (16.0-24.8)	24.7 (19.4-30.5)	20.9 (15.2-31.2)	20.3 (17.7-29.1)
*b′*	4.62 (3.82-5.26)	4.85 (4.20-5.88)	4.58 (4.08-5.38)	4.83 (3.87-5.90)	4.43 (3.78-5.36)	4.40 (3.81-5.23)	5.47 (4.65-6.31)
*c′*	2.57 (2.02-3.29)	2.20 (1.43-2.77)	2.26 (1.79-3.16)	2.01 (1.66-2.67)	1.96 (1.62-2.40)	2.27 (1.61-3.15)	2.16 (1.82-2.62)
Maximum body width	21.0 (19.4-24.9)	22.3 (20.4-28.6)	23.2 (20.1-32.4)	24.6 (19.2-30.6)	21.3 (19.4-23.4)	20.9 (18.0-27.3)	29.2 (24.9-34.8)
Esophageal length	112 (92-138)	110 (87-134)	114 (109-154)	107 (88-137)	106 (89-116)	116 (95-131)	109 (86-128)
Head end to posterior gland	131 (108-160)	129 (106-152)	132 (109-154)	126 (107-155)	124 (109-134)	134 (114-149)	129 (104-148)
Anterior end to excretory pore	94 (85-117)	92 (79-102)	90 (81-101)	86 (75-110)	87 (77-95)	88 (78-100)	95 (84-110)
Lip region height	3.17 (2.24-3.87)	3.02 (2.39-3.88)	3.02 (2.17-3.89)	2.37 (1.87-2.99)	3.04 (2.42-3.51)	3.29 (2.56-3.96)	2.43 (1.69-3.08)
Stylet	14.8 (13.8-17.7)	15.3 (14.3-16.6)	14.9 (13.6-16.6)	15.8 (14.6-17.7)	14.8 (14.0-15.9)	14.9 (13.9-15.8)	15.9 (14.8-17.4)
DGO	3.09 (2.39-4.45)	2.98 (2.21-3.88)	2.79 (2.18-3.46)	2.63 (1.77-3.32)	2.92 (2.30-3.59)	2.85 (2.11-3.57)	2.49 (1.88-3.06)
Body width at vulva	19.6 (17.4-22.5)	19.7 (17.5-24.3)	20.6 (16.7-28.9)	21.4 (16.7-25.0)	18.9 (16.4-21.6)	18.5 (15.8-22.9)	25.6 (23.0-29.2)
PUS	21.9 (11.8-35.2)	15.5 (9.2-21.0)	16.7 (11.7-16.4)	22.0 (16.3-34.9)	16.4 (11.1-23.3)	15.3 (11.3-22.5)	19.8 (13.9-28.5)
Body width at anus	12.6 (11.1-14.2)	14.0 (12.1-17.5)	14.3 (11.7-16.4)	15.1 (11.3-17.5)	13.5 (11.3-15.3)	12.8 (9.5-16.0)	16.2 (11.8-17.8)
Tail length	32.4 (26.1-44.7)	30.8 (19.4-42.6)	32.1 (27.5-42.7)	30.3 (23.1-40.3)	26.4 (21.5-31.9)	28.9 (20.2-37.9)	34.9 (25.5-43.2)
Vulva to anus distance	91 (62-124)	96.51 (86-121)	94 (75-119)	89 (73-109)	79 (65-105)	84 (65-113)	107 (90-131)
Lateral incisures	4	4 with striation	4 with striation	4	4 with striation	4 with striation	4 with striation
Labial annules	3	3	3	3	3	3	3
Tail tip^b^	SMO	SMO, ANN	SMO	SMO	SMO, CFT	SMO, ANN	SMO
Tail shape^c^	SHM, TRC, CLA	SHM, SBD, CLA	SHM	SHM	SHM, TRC, CLA	SHM, TRC	SHM
Spermatheca	Round	Round	Round	Round	Round	Round	Round
Spicule	16.2 (12.3-20.1)	15.4 (12.1-20.0)	14.4 (11.7-17.9)	14.9 (13.1-17.3)	15.3 (11.0-18.5)	15.4 (12.8-18.7)	15.2 (13.0-17.5)
Gubernaculum	3.87 (2.69-4.88)	3.98 (2.74-5.18)	3.99 (2.96-5.59)	3.75 (3.27-4.09)	3.56 (2.58-5.73)	3.60 (2.74-4.28)	4.17 (3.65-4.47)

**Notes:** ^a^ *L* = total body length; V% = vulva% from the anterior end; *a* = *L*/maximum body width; *b* = *L*/esophageal length; *c* = *L*/tail length; *b′* = *L*/anterior end to end of esophageal gland; *c′* = tail length/body width at annus; ^b^SMO = smooth, CFT = cleft, ANN = annulated; after Federick and Tarjan (1989); ^c^SHM = subhemispherical, TRC = truncate, CLA = clavate, SBD = subdigitate; after Federick and Tarjan (1989).

Mean morphometric values for the isolates were all within the range reported for *P. penetrans* by Loof (1960) except three isolates were lower than Loof’s range (5.3-7.9) for *b*, one was higher than Loof’s range (15-24) for *c*, and one was lower than Loof’s range (15-17) for stylet length (Tables 1 and 2). All isolates had four lateral incisures but individuals in seven isolates had striae in the middle band in the vicinity of the vulva. Nine isolates had a subhemispherical tail with a smooth tip. Individuals that deviated from this typical tail were detected in four isolates; the frequency of truncate tails ranged from 10 to 30% for three isolates and the frequency of annulated tail tips was ca 10% for three isolates. Males were common in all the isolates.

### Phenotype analysis of isolates

**Figure 2:**
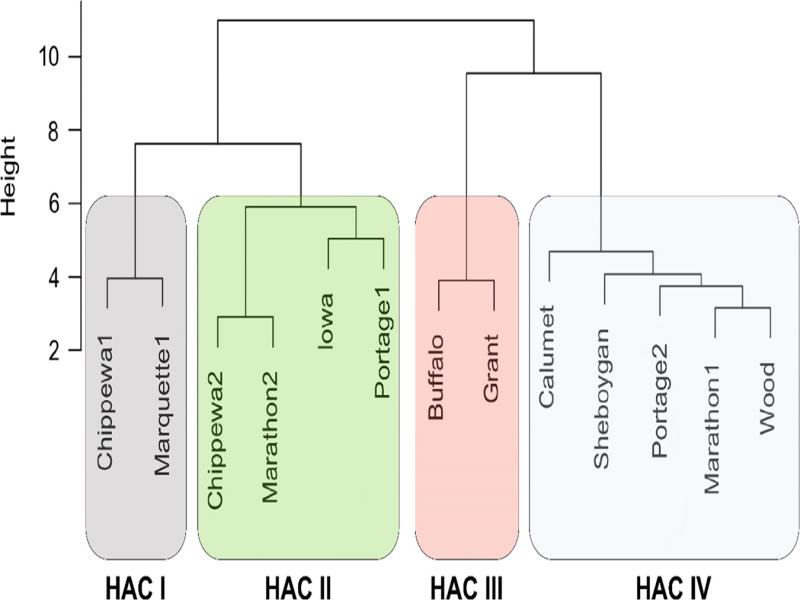
Four clusters of the Wisconsin *Pratylenchus penetrans* isolates based on morphological characters analyzed by Hierarchical Agglomerative Clustering (HAC). The scale represents the Euclidean distance in canonical space.

HAC separated the 13 *P. penetrans* isolates into four clusters based on the Euclidean distance in canonical space (Fig. 2): (I) Chippewa1 and Marquette1, (II) Chippewa2, Marathon2, Iowa and Portage1, (III) Buffalo and Grant, and (IV) Calumet, Sheboygan, Portage2, Marathon1, and Wood. There was no association between clusters and geographical origins of the isolates. Isolates in HAC clusters I and II had smooth and round tail tips, whereas both isolates in the HAC clusters III and some isolates in the HAC cluster IV (i.e. Calumet and Sheboygan) had a mix of both characters.

**Figure 3:**
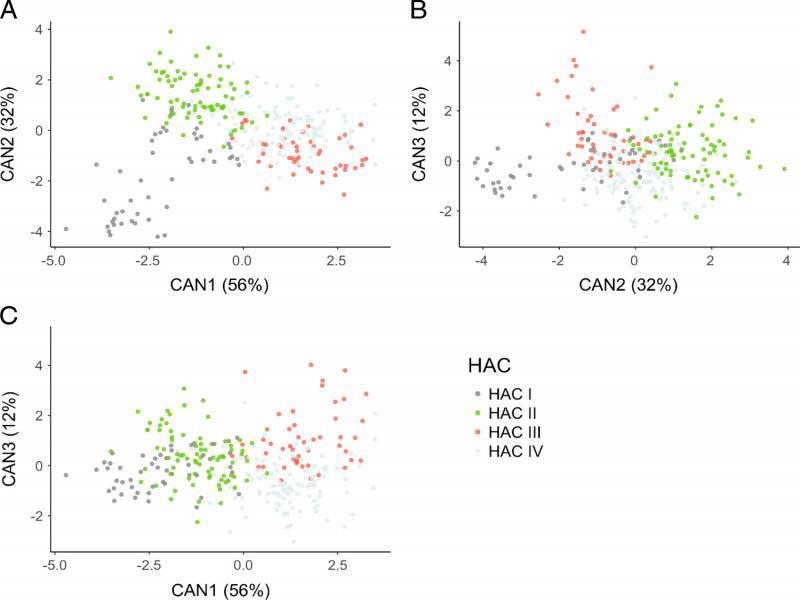
Scatter plot of the clusters of Wisconsin isolates chosen by Hierarchical Agglomerative Clustering (HAC) of morphological characters displayed using canonical variates 1 and 2 (A), canonical variates 2 and 3 (B), and canonical variates 1 and 3 (C).

The clusters determined by HAC were confirmed by the CDA analysis (Wilks’ *λ* < 0.001) (Fig. 3). The means of all the morphometrics, except for esophageal length (*P* = 0.06), were not equal among the 13 isolates (*P* < 0.01). The first two canonical variates for the CDA models explained 88% of the variability in morphometrics for clusters based on the HAC groups. The variables with the greatest influence on the discriminant functions (vector loading > 0.5) were lip region height (0.69), maximum body width (−0.61), stylet (−0.55), body width at anus (−0.53), and *a* (0.50) for the first canonical variate; maximum body width (0.64), total body length (0.61), length from anterior end to excretory pore (0.52) for the second canonical variate; and vulva percentage (0.71) and tail length (0.54) for the third canonical variate. There was reasonable separation of clusters I, II, and III, but the distinction of cluster IV from the other clusters was not resolved (Fig. 3).

### Molecular characterization

The 402 bp of nucleotide sequence was read without gaps by the COI primer. The pairwise sequence identity between the 13 Wisconsin isolates ranged from 94.2% (Portage1 and Marathon2) to 100% similarity (Table 4). There were 30 single nucleotide polymorphisms (SNPs) on COI sequences among the 13 *P. penetrans* isolates. The most common mutations were between C and T. Similar mutations were also observed at the same sites among the COI sequences of our field populations and those obtained from GenBank. The largest nucleotide divergence in COI between a Wisconsin population and a population elsewhere was 92.7% for Sheboygan-a and the T716 population (Stramproy) from the Netherlands (Janssen et al., 2017), but the species identification of the Dutch population was not confirmed by a gene in addition to COI. The largest nucleotide divergence in COI with nematodes confirmed to be *P. penetrans* by 28S rDNA was between Marathon2-a and the V1B population (Meijel) from the Netherlands (Janssen et al., 2017), at 94.5% similarity. There was no variation in amino acid sequences of *P. penetrans* populations as a result of SNPs on COI.

**Table 4.** Pairwise distance (percent similarity) for CO1 mtDNA between 13 *P. penetrans* isolates from Wisconsin.

Population	Portage 1	Portage 2	Marathon 1	Marathon 2	Chippewa 1	Chippewa 2	Calumet	Sheboygan	Wood	Marquette	Buffalo	Grant	Iowa
*% similarity on COI sequences*													
Portage 1													
Portage 2	99.5												
Marathon 1	94.5	95.0											
Marathon 2	94.2	94.8	99.8										
Chippewa 1	95.0	95.0	95.5	95.5									
Chippewa 2	94.5	95.0	100.0	100.0	95.5								
Calumet	94.5	95.0	100.0	100.0	95.5	100.0							
Sheboygan	95.0	95.0	95.5	95.5	99.5	95.5	95.5						
Wood	95.5	95.5	96.5	96.5	98.5	96.5	96.5	98.5					
Marquette	94.5	95.0	100.0	100.0	95.5	100.0	100.0	95.5	96.5				
Buffalo	94.5	95.0	100.0	100.0	95.5	100.0	100.0	95.5	96.5	100.0			
Grant	94.5	95.0	100.0	100.0	95.5	100.0	100.0	95.5	96.5	100.0	100.0		
Iowa	94.5	95.0	100.0	100.0	95.5	100.0	100.0	95.5	97.6	100.0	100.0	99.9	

### Haplotype network analysis

**Figure 4:**
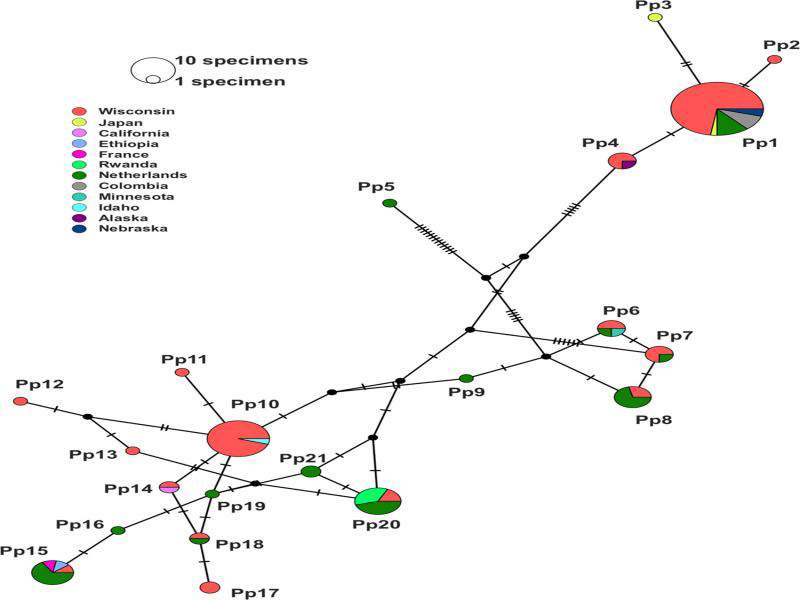
COI (mitochondrial cytochrome c oxidase subunit 1) haplotype network of *Pratylenchus penetrans* using TCS network. Circle size and hash marks between haplotypes represent numbers of specimens included in each haplotype and mutations, respectively. Geographical location of the population is designated by color. Median vectors are shown as black circles.

A total of 120 sequences, 72 from Wisconsin, revealed 21 haplotypes for *P. penetrans* based on COI (Fig. 4). The Wisconsin populations were assigned to 15 of the 21 haplotypes: three shared with other populations from the USA, seven shared with populations from other countries, and five that were unique to Wisconsin. The most common haplotype in Wisconsin was Pp1, which included 32 Wisconsin populations as well as populations from Nebraska, USA, Colombia, The Netherlands, and Japan (Fig. 4). The second most frequent haplotype in Wisconsin, Pp10, included 19 Wisconsin populations and one population from Idaho, USA. In 17 cases, multiple specimens from the same population were assigned to different haplotype groups (Table 3). No relationship was observed between haplotype and HAC assignment for the 13 Wisconsin isolates.

### Phylogenetic analysis

**Figure 5:**
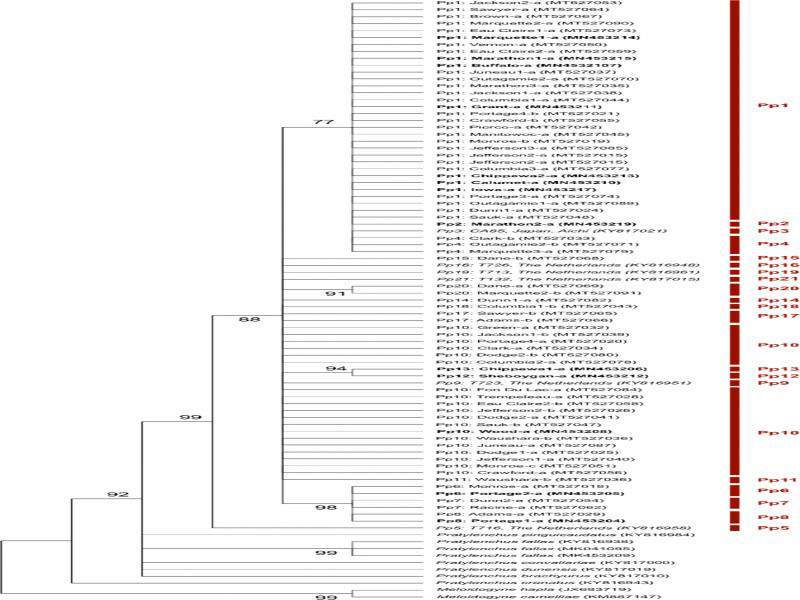
Maximum likelihood tree of mitochondrial cytochrome c oxidase subunit 1 (COI) sequences to infer evolutionary relationship of the Wisconsin *Pratylenchus penetrans* populations. Bootstrap values below 75 were omitted from the nodes. Haplotypes are labeled and also designated by the vertical red bar. Information about the haplotypes appears in Table 3.

The ML tree based on COI validated that the 72 Wisconsin populations were conspecific with *P. penetrans* (Fig. 5). The bootstrap value supported the assignment of *P. penetrans* populations to a clade distinguished from the other species in the Penetrans group and *P. crenatus.* Within the *P. penetrans* clade, T716, the Dutch population from Janssen et al. (2017), was assigned to a sub-clade that was distant from all others, but it should be noted that species confirmation by 28S rDNA or other genes is not available for that population. Haplotypes Pp1 to Pp4 were distinguished from others and grouped together in one clade, as were Pp6 to Pp8. The evolutionary relationship of other haplotypes was not clearly resolved in the analysis.

## Discussion

Our study demonstrated considerable intraspecific variation in morphological and molecular characters for *P. penetrans* populations from Wisconsin that was not associated with geography. Haplotype analysis showed differences among individuals collected from the same field population, as was the case for *P. penetrans* populations in the Netherlands (Janssen et al., 2017). Cultured isolates originating from samples collected in the same county (Chippewa) also differed for haplotype and showed morphological variation detected by HAC analysis. Intraspecific variation at local scales was juxtaposed to similarity in haplotypes that spanned continents. Wisconsin populations shared the same haplotypes as populations from North America, South America, Europe, Asia and Africa. Populations of *P. penetrans* from the Netherlands were sympatric for COI with populations from Europe, South America, Asia, and Africa (Janssen et al., 2017), so this is the first report of *P. penetrans* COI haplotypes shared by Europe and North America. Six haplotypes unique to the Netherlands (Janssen et al., 2017) were consistent with our analysis and we detected five haplotypes unique to Wisconsin, so it is likely that even more intraspecific variability will be revealed as more populations of *P. penetrans* are sequenced.

Three morphological features used to distinguish *P. penetrans* as a species, three lip annuli, a round spermatheca filled with sperm (i.e. presence of males), and four lines in the lateral field, were robust for our isolates. Most keys for identifying *Pratylenchus* spp. assign a smooth tail tip to *P. penetrans* (Loof, 1960; Corbett, 1970; Handoo and Golden, 1989; Castillo and Vovlas, 2007), but we and others (Taylor and Jenkins, 1957; Tarte and Mai, 1976a; Townshend, 1991; Rusinque et al., 2020) noted some individuals with annulated tail tips. Tarte and Mai (1976a) demonstrated that the frequency of annulated tail tips was influenced by the plant host, but they also found the only case of exclusively smooth tail tips in an isolate started with a single smooth-tailed female (Tarte and Mai, 1976b). They speculated that tail tip phenotype is at least partly under genetic control. Nine of our isolates had a 100% frequency of the smooth tail tip phenotype, but four did not and all were reared *in vitro* in the same stable environment, supporting the possibility that tail tip phenotype is determined genetically.

Data for 49 isolates and populations of *P. penetrans* (Sher and Allen, 1953; Taylor and Jenkins, 1957; Loof, 1960; Román and Hirschmann, 1969; Tarte and Mai, 1976a; Vovlas and Troccoli, 1990; Townshend, 1991; Troccoli et al., 1992; Ozbayrak, 2019; Ryss, 2002; Mekete et al., 2011; Mokrini et al., 2016; Janssen et al., 2017; Rusinque et al., 2020), including our study, provided opportunity to review morphometrics of the species presented in seminal publications by Sher and Allen (1953) and Loof (1960). Loof’s range for V% (75-84) was a closer fit to the data (*n* = 39) than that of Sher and Allen (78-83). The minimum mean value for V% was 75% shared by two isolates from Morocco (Mokrini et al., 2016) and the maximum was 82% for a population from Nebraska (Ozbayrak, 2019). In total, 49% of the isolates and populations had a mean value for V% of less than 80%, suggesting that the range of 80 to 85% used in a recent tabular key (Castillo and Vovlas, 2007) is not representative of many *P. penetrans*, including three isolates from Wisconsin. The smallest value for mean *a* index (*n* = 46) was 21.1 in a population from Algeria (Troccoli et al., 1992) and the maximum was 33.1 in two isolates from Morocco (Mokrini et al., 2016), which was above the maximum set by Loof (31.9) and Sher and Allen (30). The largest mean value of the *b* index (*n* = 43) was within the maximum set by Loof (7.9), but well above the maximum of Sher and Allen (6.5) and the minimum value was 4.5 for a population from the Netherlands (Janssen et al., 2017) which was below the minimum of both Loof (5.3) and Sher and Allen (5.7), as were three Wisconsin isolates. Three isolates (*n* = 46), one each from Wisconsin, Morocco (Mokrini et al., 2016) and the Netherlands (Janssen et al., 2017), had a mean value for the *c* index that exceeded the upper limit published by Loof (23.8) and Sher and Allen (21). Seven Wisconsin isolates and a population from Canada (Townshend, 1991) had a mean stylet length below 15 μm and one population from Portugal had a mean stylet length of 18.5 μm (Rusinque et al., 2020), falling outside the maximum published by Loof (17 μm) and the minimum published by both Loof (15 μm) and Sher and Allen (17 μm). Most of the populations outside of the commonly recognized ranges were identified using molecular as well as morphological criteria, so they are likely to be valid *P. penetrans* which suggests there is even more intraspecific variability within the species than previously considered.

We detected enough pattern in morphological variation to assign our 13 Wisconsin isolates of *P. penetrans* to four clusters. Two features, stylet length and height of the cephalic lip region, showed less variation within than among isolates and were identified as important by CDA analysis. Contrary to our results, these features were similar among 13 Moroccan *P. penetrans* isolates reared *in vitro* (Mokrini et al., 2016). Based on the relatively weak separation of some of our clusters and the possibility that our results are idiosyncratic to Wisconsin, we do not consider our clusters to represent morphotypes.

Our haplotype analysis revealed up to 5.8% divergence in COI DNA among populations confirmed to be *P. penetrans* by multiple genes. As was the case in the Netherlands (Janssen et al., 2017), the nucleotide divergence did not change amino acid sequences, confirming that they were still conspecific with *P. penetrans*. Our ML tree showed the same four groups presented by Janssen et al. (2017) with Wisconsin isolates assigned to three (i.e., Janssen’s A, B, and C) groups. The most common haplotype in Wisconsin, Pp1, was the second most common haplotype of the Dutch populations (Pe11) and included populations from South America. The second most common haplotype in Wisconsin, Pp10, was comprised exclusively of *P. penetrans* populations from the northern USA. In total, 11 haplotypes were unique to a single country. A gradient ranging from cosmopolitan to localized distribution suggests that dispersal events for *P. penetrans* are frequent and ongoing.

The widespread incidence and high degree of intraspecific variability of *P. penetrans* coupled with the state’s edaphic and agricultural diversity makes Wisconsin an interesting place to study this cosmopolitan plant pest. The success of *P. penetrans* is most likely due to its life history traits and agricultural activities. The species, highly polyphagous and capable of long-term survival in an anhydrobiotic state (Townshend, 1984), was likely introduced to Wisconsin in vegetatively propagated crops such as potato or in grain contaminated with soil peds. It would be interesting to compare Wisconsin and German isolates, as 37% of the state’s citizens in 1890 originated from Germany and most were farmers (Wyman, 1968). There are many approaches and characters useful for studying intraspecific variability for nematodes. Our results echo previous studies (Janssen et al., 2017; Ozbayrak et al., 2019) in demonstrating the utility of the COI mtDNA gene for distinguishing populations of *P. penetrans* as well as their relationships.
